# RNA binding proteins in cancer chemotherapeutic drug resistance

**DOI:** 10.3389/fcell.2024.1308102

**Published:** 2024-01-24

**Authors:** Hemanathan Vembuli, Ravi Gor, Satish Ramalingam, Selene Perales, Johnson Rajasingh

**Affiliations:** ^1^ Department of Genetic Engineering, School of Bio-Engineering, SRM Institute of Science and Technology, Kattankulathur, Tamil Nadu, India; ^2^ Department of Bioscience Research, University of Tennessee Health Science Center, Memphis, TN, United States; ^3^ Department of Microbiology, Immunology, and Biochemistry, University of Tennessee Health Science Center, Memphis, TN, United States

**Keywords:** RNA binding proteins, cancer, drug resistance, cancer stem cells, miRNA, and lncRNA

## Abstract

Drug resistance has been a major obstacle in the quest for a cancer cure. Many chemotherapeutic treatments fail to overcome chemoresistance, resulting in tumor remission. The exact process that leads to drug resistance in many cancers has not been fully explored or understood. However, the discovery of RNA binding proteins (RBPs) has provided insight into various pathways and post-transcriptional gene modifications involved in drug tolerance. RBPs are evolutionarily conserved proteins, and their abnormal gene expression has been associated with cancer progression. Additionally, RBPs are aberrantly expressed in numerous neoplasms. RBPs have also been implicated in maintaining cancer stemness, epithelial-to-mesenchymal transition, and other processes. In this review, we aim to provide an overview of RBP-mediated mechanisms of drug resistance and their implications in cancer malignancy. We discuss in detail the role of major RBPs and their correlation with noncoding RNAs (ncRNAs) that are associated with the inhibition of chemosensitivity. Understanding and exploring the pathways of RBP-mediated chemoresistance will contribute to the development of improved cancer diagnosis and treatment strategies.

## Introduction

**FIGURE 1:**
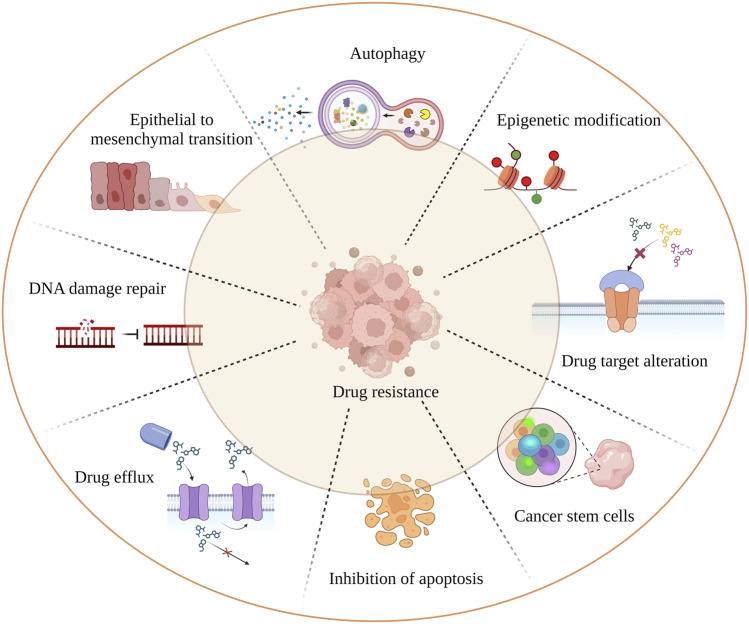
Mechanism of tumor drug resistance. The hall marks of cancer drug resistance such as EMT; cancer stem cell population; failure of apoptosis; activation of autophagy leading to cancer cell death inhibition; spontaneous DNA damage repair; rapid drug detoxification by drug efflux and target modification; are the underlying factors which cause cancer remission and treatment failure.

Cancer remains one of the leading causes of mortality worldwide. Despite advances in chemotherapeutic strategies, drug resistance continues to hinder progress towards a cure. Reports indicate that 90% of drug resistance occurs during tumor invasion and metastasis (Dreyfuss et al., 2002; Mansoori et al., 2017). While much research has focused on intrinsic resistance (pre-existent) and acquired resistance (induced by drugs), tumors can inherit a combination of these resistance mechanisms (Vasan et al., 2019). The independent and combined effects of mechanisms such as drug inactivation, drug target alteration, drug efflux (ABC transporters), DNA damage repair, inhibition of cell death, epithelial to mesenchymal transition (EMT), cancer cell heterogeneity, and epigenetic modification (DNA methylation, histone modification, *etc.*) significantly reduce the effectiveness of cancer drugs (Housman et al., 2014) (Figure 1). In addition, targeting the cancer stem cells (CSCs) population is crucial as it promotes tumor differentiation, self-renewal, and invasiveness. Most chemotherapeutic drugs fail because they are unable to eliminate CSCs. To overcome resistance to monotherapy, combination therapy has been adapted and proven to be effective in reducing tumor burden and remission (Mokhtari et al., 2017). However, combination therapy may also increase toxicity to normal cells. Therefore, a novel therapeutic approach is needed to overcome drug resistance. Recent research has found a link between drug resistance and aberrant expression of RNA binding proteins (RBPs), suggesting that RBPs could be potential targets for developing anti-chemoresistance therapies. This review focuses on RBPs, particularly HuR, NONO, IGF2BP2 and 3, Musashi1 and 2, Lin28, RBM, Pumilio, YTHDF1, and AUF1, which are most extensively studied in association with cancer drug resistance. We also addressed the link between RBPs and miRNAs, which play a major role in chemoresistance (Kim et al., 2021a). We intend to provide insight on current findings on the relationship between RBPs and cancer chemoresistance, as well as to highlight noteworthy *in vivo* (Table 1) and cancer clinical research studies (Table 2) that target RBPs.

**TABLE 1:** List of animal studies employed in RBP mediated chemoresistance.

S.no	RBP	Animal models	Cancer type	Drug/Treatment	References
1	Lin28	BALB/c mice	Gastric cancer	Doxorubicin, Oxaliplatin	Teng et al. (2015)
2	HuR	Female Ncr nu/nu mice	Pancreatic cancer		Dong et al. (2020)
3	HuR	Male BALB/cA-nu mice	Colorectal cancer	Oxaliplatin	Cai et al. (2019)
4	HuR	BALB/c nu/nu male athymic nude mice	Colorectal cancer	Oxaliplatin	Deng et al. (2020)
5	HuR	Athymic nude mice	Oral cancer	Paclitaxel	Janakiraman et al. (2017)
6	HuR	Mice	Glioblastoma		Filippova et al. (2011)
7	NONO	Female BALB/c nude mice	Triple negative breast cancer		Kim et al. (2020)
8	NONO	Female NOD/SCID mice	Acute myelogenous leukemia		Zhang et al. (2022a)
9	NONO	Male nude mice	Melanoma		Zhang et al. (2021a)
10	NONO	Nude mice	Gallbladder carcinoma	Gemcitabine	Xue et al. (2020)
11	IGF2BP1	NSG mice	Leukemia		Elcheva et al. (2020)
12	IGF2BP2	Female nude mice	Ovarian cancer	Cisplatin	Fu et al. (2022)
13	IGF2BP3	Male nude mice	Colorectal cancer		Xu et al. (2019)
14	IGF2BP3	BALB/c nude mice	NSCLC	EGFR inhibitors	Lin et al. (2023)
15	MSI1	Immune compromised mice	Glioblastoma		Chen et al. (2018)
16	MSI2	Immunocompromised NSG mice	Lung adenocarcinoma	Osimertinib	Yiming et al. (2021)
17	MSI2	Male nude mice	Hepatocellular carcinoma		Fang et al. (2017)
18	RBMX	Female nu/nu mice	Hepatocellular carcinoma	Sorafenib	Song et al. (2020)
19	Pumilio	Male or female BALB/c nude mice	Colorectal cancer		Gong et al. (2022)
20	YTHDF1	Female BALB/c nude mice	Breast cancer	Adriamycin, cisplatin, olaparib	Sun et al. (2022)
21	YTHDF1	Nude mice (BALB/c)	Colorectal cancer	Cisplatin	Chen et al. (2021a)
22	YTHDF1	Nude mice (BALB/c)	Gastric cancer		Chen et al. (2021b)
23	AUF1	Male NPG/Vst mice	Hepatocellular carcinoma	Doxorubicin	Zhang et al. (2022b)
24	AUF1	Female nude mice	Breast cancer	Cisplatin, docetaxel	Al-Tweigeri et al. (2022)
25	MSI1	Female C57BL/6 mice	Acute myelogenous leukemia	small molecule Ro 08–2,750 (Ro)	Gor et al. (2023)

**TABLE 2:** Various RBPs and the drugs employed in clinical studies.

S.no	RBP	Number of patients	Cancer type	Drug/Treatment	References
1	Lin28	9	Breast cancer	Paclitaxel	Lv et al. (2012)
2	HuR	82	Breast cancer		Zhu et al. (2013)
3	HuR	160	Colorectal cancer	Oxaliplatin	Cai et al. (2019)
4	HuR	26	Colorectal cancer		To et al. (2015)
5	HuR	31	NSCLC		Gong et al. (2019)
6	HuR	30	Colorectal cancer	Oxaliplatin	Deng et al. (2020)
7	NONO	10	Bladder cancer		Qin et al. (2021)
8	NONO	110	Gallbladder carcinoma	Gemcitabine	Xue et al. (2020)
9	IGF2BP2	62	Ovarian cancer	Cisplatin	Fu et al. (2022)
10	IGF2BP3	192	Colorectal cancer		Xu et al. (2019)
11	IMP3	482	Renal cell carcinoma		Pei et al. (2015)
12	IGF2BP3	236	Hepatocellular carcinoma	Isocorydine (d-ICD)	Li et al. (2015)
13	MSI1	3	Colorectal cancer		Chiou et al. (2017)
14	MSI1	79	NSCLC		Lang et al. (2017)
15	Lin28	69	NSCLC		Yin et al. (2017)
16	RBM3	307	Bladder cancer	Cisplatin and gemcitabine	Wahlin et al. (2022)
17	RBMX	60	Hepatocellular carcinoma	Sorafenib	Song et al. (2020)
18	RBM38	10	Breast cancer	Adriamycin	Ke et al. (2022)
19	Pumilio-2	20	NSCLC	Cisplatin	Zhang et al. (2021b)
20	YTHDF1	50	Colorectal cancer	Cisplatin	Chen et al. (2021a)
21	AUF1	66	Hepatocellular carcinoma	Doxorubicin	Zhang et al. (2022b)
22	AUF1	344	Breast cancer	Cisplatin, docetaxel	Al-Tweigeri et al. (2022)

### RNA binding proteins (RBPs)

**FIGURE 2:**
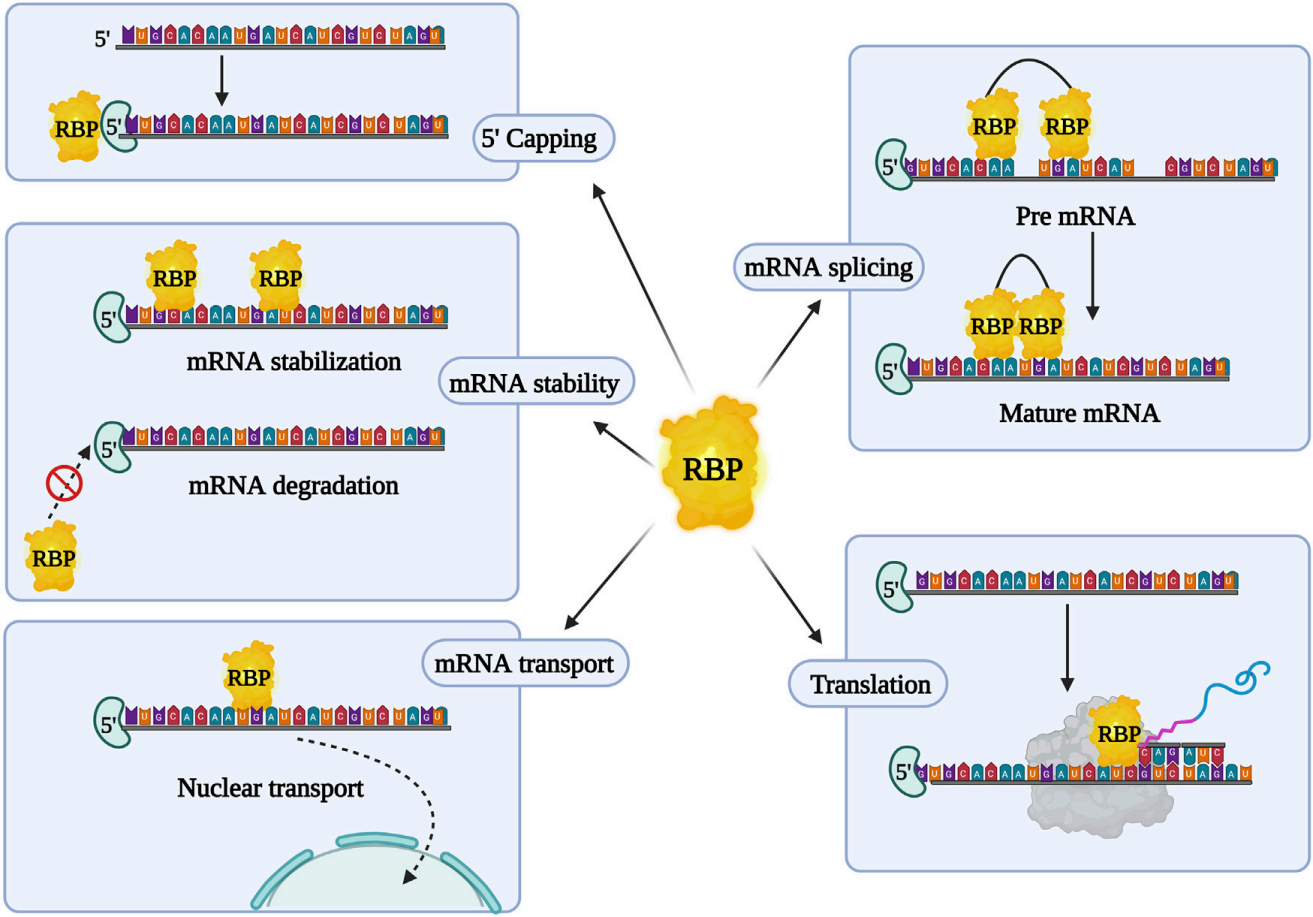
Overview of RNA binding proteins (RBPs) and their characteristic features. RBPs aid in post transcriptional regulation and initiation or suppression of mRNA translation.

RBPs form complexes with mRNA and regulate gene expression post-transcriptionally, by creating ribonucleoprotein (RNP) complexes. They bind to the protein binding domain on the 3′ or 5′ untranslated region (UTR), coding regions of the mRNA, and non-coding RNA (ncRNA) to control gene expression (Gerstberger et al., 2014). RNA and RBPs exhibit cooperative effects as they influence each other’s functions. RBPs control RNA via miRNA through post-transcriptional modifications in the nucleus (transcription, splicing, capping, and polyadenylation) and in the cytoplasm (mRNA transport, localization, degradation, repression, stabilization, and translation activation) (Figure 2). Conversely, RNAs and ncRNAs can also modulate the stability, localization, interaction, and functions of RBPs (Thelen and Kye, 2020; Gebauer et al., 2021). Abnormal profiles of RBPs regulate various mRNAs, contributing to the occurrence and development of various malignant tumors and chemoresistance (Table 3). In cancer, dysregulated RBPs can affect their target mRNA transcripts, promoting cancer proliferation, angiogenesis, invasion, metastasis, and inhibition of apoptosis (Thelen and Kye, 2020). Several RBPs have been studied for their correlation between chemoresistance and aberrant expressions, such as the higher expression of Lin28 in gastric, breast, and colon cancer (Lv et al., 2012; Teng et al., 2015), and others. Furthermore, RBPs have a positive and negative regulatory effect on miRNA targeting (MT). Increased accessibility to MT is consistent with the shortest distance between the miRNA target site and the closest RBP on the 3′UTR. To add on, miRNAs loaded into Argonaute (AGO) proteins largely substituted RBPs linked to the target mRNA, resulting in elevated MT. Experimental validation by Kim’s research group demonstrated that hampering RBPs significantly lowered MT. As a result, irregular RBP expression in cancer patients may contribute to the development of chemoresistance in cancer cells via deficient miRNA targeting. The interlink between ncRNA and RBPs pertaining to chemoresistance has been listed in Table 4. Overall, there are several mechanisms involved in RBP expression regulation, and the effect of ncRNA-RBP interaction contributes to fine-tuning RBP activity. Identifying the molecular pathways behind chemoresistance is a major focus of cancer research, and RBPs hold considerable promise as prospective targets for chemoresistance modulation.

**TABLE 3:** RBPs and their interactions with target mRNA show chemoresistance modulation in various cancer.

No.	RBP	Target mRNA	Target mRNA function	Drug sensitive/resistance	Drugs	Cancer type	References
1	HUR ↑	CDC6 ↑	DNA pre-replication complex	Resistance	Oxaliplatin	Colon cancer	Cai et al. (2019)
2	HUR ↑	ERBB2 ↑	Receptor tyrosine-protein kinase	Resistance	Tamoxifen	Breast cancer	Tan et al. (2017)
3	HUR ↓	Nil		Sensitive	Epirubicin	Colon cancer	Lin et al. (2017)
4	HUR ↓	Nil		Sensitive	Etoposide, Topotecan and Cisplatin	Glioma	Filippova et al. (2011)
6	NONO ↑	STAT3 ↑	Transcription factor, activates anti-apoptotic and immune related genes	Resistance	Doxorubicin	Triple-negative breast cancer	Kim et al. (2020)
7	NONO ↑	STAT3, SPHK1 ↑	Cell differentiation, motility, and apoptosis	Resistance	Cisplatin	Bladder cancer	Qin et al. (2021)
8	NONO ↑	SAMHD1 ↑	Regulation of innate immune response	Resistance	Cytarabine	Acute myelogenous leukemia	Zhang et al. (2022a)
9	NONO ↑	pERK1/2 ↑	Cell proliferation regulator	Resistance	AZ628 and Vemurafenib	Triple-negative breast cancer	Zhang et al. (2021a)
10	IGF2BP2 ↓	Nil		Sensitive	Pemetrexed	Non-small cell lung cancer	Wang et al. (2021)
11	IGF2BP1 ↓	Nil		Sensitive	Doxorubicin, Cytarabine and Cyclophosphamide	Leukemia	Elcheva et al. (2020)
12	IGF2BP3 ↑	ABCG2 ↑	Multi drug resistance	Resistance	Doxorubicin and mitoxantrone	Triple-negative breast cancer	Samanta et al. (2013)
13	IGF2BP3 ↑	COX6B2 ↑	Oxidative phosphorylation	Resistance	tyrosine kinase inhibitors	Non-small cell lung cancer	Lin et al. (2023)
14	MSI2 ↑	NANOG ↑	Transcription factor, pluripotency maintenance	Resistance	Gefitinib, Osimertinib	Non-small cell lung cancer	Yiming et al. (2021)
15	MSI2 ↓	Nil		Sensitive	Daunorubicin	Acute Myeloid Leukemia	Han et al. (2015)
16	MSI2 ↓	Nil		Sensitive	Sorafenib	Hepatocellular carcinoma	Fang et al. (2017)
17	YTHDF1 ↑	E2F8 ↑	Transcription factor	Resistance	Cisplatin, Adriamycin and PARP inhibitor Olaparib	Breast cancer	Sun et al. (2022)
18	YTHDF1 ↑	GLS1 ↑	Transforms glutamine into glutamate by hydrolysis	Resistance	Cisplatin	Colon cancer	Chen et al. (2021a)
19	AUF1 ↑	AKR1B10 ↑	Aldo-keto reductase	Resistance	Doxorubicin	Hepatocellular carcinoma	Zhang et al. (2022b)
20	AUF1 ↑	Nil		Resistance	Cisplatin and Docetaxel	Advanced breast cancer	Al-Tweigeri et al. (2022)

**TABLE 4:** Crosstalk between RBPs and ncRNA in regulating chemoresistance.

S.no	RBP	Target ncRNA	Drug sensitive/resistance	Drugs	Cancer type	References
1	Lin28 ↑	miR-107 ↓	Resistance	Oxaliplatin, Paclitaxel, Doxorubicin, Fluorouracil	Gastric cancer	Teng et al. (2015)
2	Lin28 ↑	Let-7 ↓	Resistance	Paclitaxel	Breast cancer	Lv et al. (2012)
3	HUR ↓	miR133 ↑	Sensitive	Docetaxel	Prostate cancer	Liu et al. (2018)
4	HUR ↑	CCAL ↑	Resistance	Oxaliplatin	Colon cancer	Deng et al. (2020)
5	HUR ↓	LINC-PINT ↑	Sensitive	Paclitaxel	Triple-negative breast cancer	Chen et al. (2020)
6	NONO ↑	SSTR5-AS1 ↑	Resistance	Gemcitabine	Gallbladder carcinoma	Xue et al. (2020)
7	IGF2BP2 ↑	DANCR ↑	Resistance	Etoposide	Glioblastoma	Han et al. (2022)
8	IGF2BP2 ↑	TUG1 ↑	Resistance	Cisplatin	Colon cancer	Xia et al. (2022)
9	IGF2BP2 ↓	miR-129-5p ↑	Sensitive	Temozolomide	Glioblastoma	Wang et al. (2022)
10	IGF2BP2 ↑	OIP5-AS1 ↑	Resistance	Temozolomide	Glioblastoma	Wang et al. (2022)
11	IGF2BP2 ↑	miR-96-5p ↑	Resistance	Cisplatin	Cervical cancer	Wu et al. (2022)
12	IGF2BP2 ↓	TRIM52-AS1 ↑	Sensitive	Cisplatin	Cervical cancer	Wu et al. (2022)
13	IGF2BP2 ↑	circPBX3 ↑	Resistance	Cisplatin	Ovarian cancer	Fu et al. (2022)
14	MSI1 ↓	miR-181a-5p ↑	Sensitive	Cisplatin	Small Cell Lung Carcinoma	Lang et al. (2017)
15	RBMx ↑	LncBLACAT1 ↑	Resistance	Sorafenib	Bladder cancer	Song et al. (2020)
16	RBM38 ↓	miR320b ↑	Sensitive	Adriamycin	Breast cancer	Ke et al. (2022)

#### HuR

Human antigen R (HuR) is an RNA-binding protein and a member of the embryonic lethal abnormal vision (ELAV) family. HuR was initially identified as crucial for neuronal development in *Drosophila* (Campos et al., 1985). HuR binds to adenine and uracil-rich elements (AREs) on the 3′UTR of the target mRNAs through RNA recognition motifs (RRM1, RRM2, and RRM3). High affinity recognition motifs such as AUUUA, AUUUUA, and AUUUUUA are critical for efficient binding (Ma et al., 1996). Normal cells predominantly express HuR in the nucleus, while cancer cells exhibit abundant cytoplasmic HuR expression. This translocation of HuR between the nucleus and the cytoplasm is facilitated by the presence of the HuR nucleocytoplasmic shuttling sequence (HNS), which contains both nuclear localization signal (NLS) and nuclear export signal (NES) sequences (Fan and Steitz, 1998). A clinical investigation of eighty-two breast cancer patients revealed elevated cytoplasmic HuR levels (Zhu et al., 2013). It was found that HuR was the primary cause of tamoxifen resistance in breast cancer. HuR accumulated more in the cytoplasm when tamoxifen was exposed to estrogen receptor-positive cells over an extended period of time. However, it has also been demonstrated that activation of MAPKs raises cytoplasmic HuR levels. In response, the Hostetter group employed a combination of tamoxifen and a JNK inhibitor, which dramatically reduced the levels of cytoplasmic HuR and caused apoptosis in the tamoxifen-resistant BT474 cell line (Hostetter et al., 2008). This study precisely showed that HuR nucleocytoplasmic shuttling was inhibited by combinatorial treatment. Conversely, in MCF7 breast cancer doxorubicin-resistant cells, reduced expression and minimal cytoplasmic translocation of HuR led to a reduction in doxorubicin-induced apoptosis (Latorre et al., 2012). These two investigations strongly emphasized the crucial role of HuR shuttling in chemoresistance.

HuR overexpression has been reported to play an active role in enhancing EMT, a hallmark of drug resistance, by upregulating mesenchymal markers such as vimentin and snail and downregulating epithelial markers. In addition, both HuR knockdown and siRNA-mediated silencing have been shown to reduce the formation of spheroids when compared to the control (Dong et al., 2020). HuR upregulation positively regulates CDC6 expression by directly binding to its 3′UTR, leading to colon cancer progression. CDC6 has been strongly associated with HuR-mediated oxaliplatin resistance. Silencing of CDC6 has been found to restore sensitivity to oxaliplatin in HuR overexpressing cells by reducing drug resistance hallmarks such as cancer cell migration, invasion, and EMT (Cai et al., 2019). These findings strongly suggest that HuR overexpression promotes chemoresistance in a variety of cancer types, although the molecular mechanisms/pathways involved in chemoresistance remain unknown. As previously indicated, RBPs influence miRNA targeting; in this case, HuR also regulates multiple miRNA targeting. For example, paclitaxel-resistant prostate cancer (PC3PR) cells exhibited low levels of miR-34a. An investigation by the Kojima group revealed that paclitaxel-resistant cells had higher HuR expression than non-resistant cells, and this adversely impacted miR-34a targeting. This suppression of miR-34a led to overexpression of tumor promoter gene SIRT1 and the anti-apoptotic gene BCL2, thereby conferring paclitaxel resistance (Kojima et al., 2010). Additionally, the Kenneth team’s clinical research showed that miR-519c mimics inhibited the HuR/ABCG2 axis, increasing the sensitivity of 5-FU to 5-FU-resistant colon cancer cells (To et al., 2015). This finding opens up the possibility that miRNA could be employed to overcome drug resistance. Surprisingly, HuR overexpression improved the doxorubicin sensitivity of cancer cells via promoting topoisomerase IIα (TOP2A) expression (Srikantan et al., 2011). Furthermore, the interaction between HuR and miR-133b has been observed in prostate cancer cells. Overexpression of HuR in these cells contributes to chemoresistance to docetaxel (DTX). Conversely, overexpression of miR133 suppresses HUR expression, thereby inhibiting the drug efflux protein ABCG2. This novel pathway involving HUR/CD133 and ABCG2 may offer a potential avenue to restore sensitivity to DTX in prostate cancer cells (Liu et al., 2018). Similarly, miR26a/b and HuR regulate the expression of Erb-B2 receptor tyrosine kinase 2 (ERBB2). HuR binds to the 3′UTR of ERBB2 and positively impacts its expression by increasing the stability of the transcripts, while upregulation of miR26a/b represses ERBB2 expression, thereby conferring higher sensitivity to tamoxifen in MCF7 breast cancer cell lines. Furthermore, cleavage stimulation factor subunit 2 (CSTF2) has been found to assist in alternative polyadenylation-induced shortening of the 3′UTR of HuR mRNA, which is essential for increased HuR translation in tamoxifen-resistant (TAMR) estrogen receptor-positive breast cancer (Tan et al., 2017).

**FIGURE 3:**
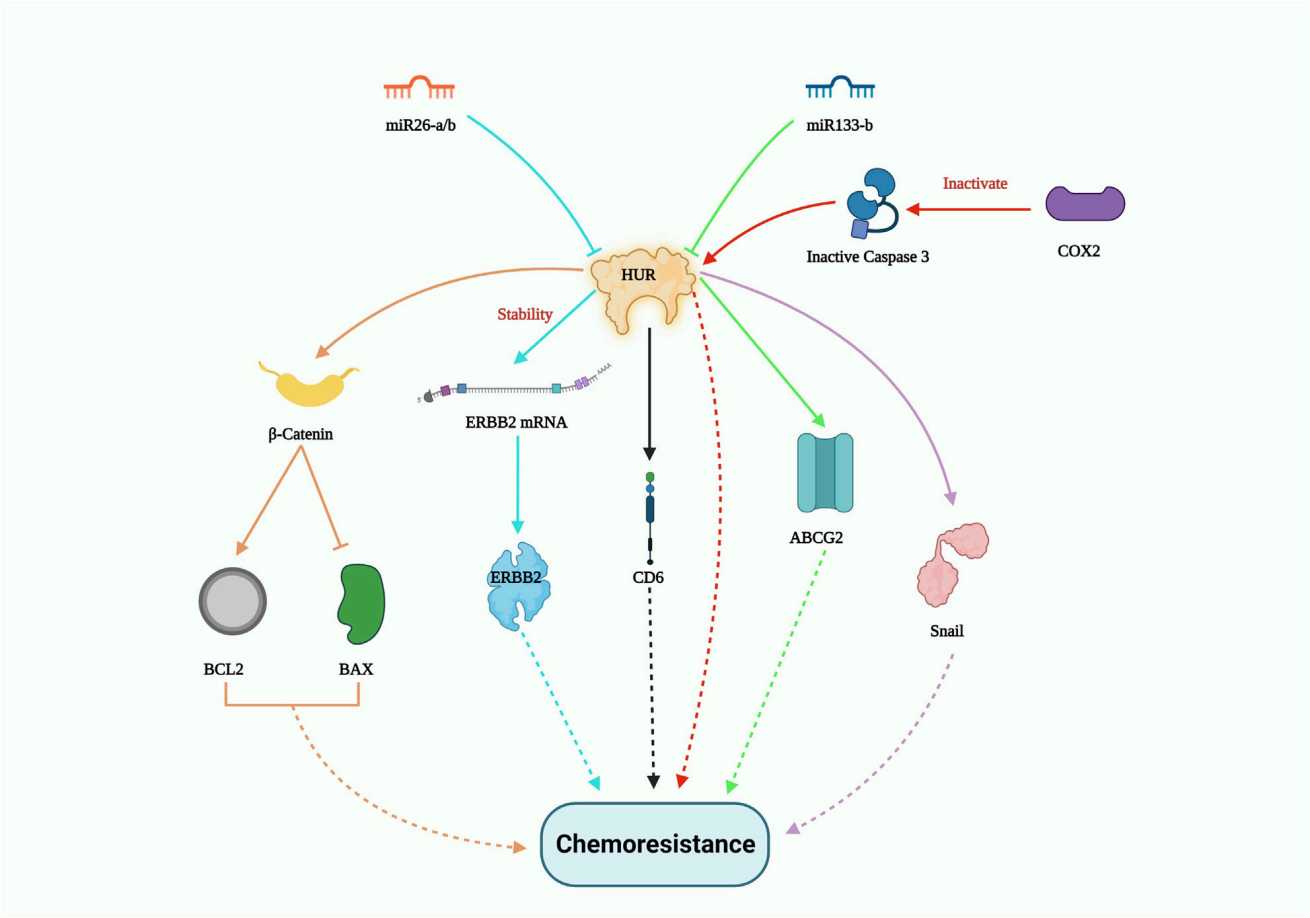
HUR-mediated drug resistance pathway. HUR activates certain genes such as β-catenin; ERBB2; CDC6; ABCG2 and Snail to induce chemoresistance, however miR26-a/b and miR133-b potentially controls HUR expression levels and thus could pave way to inhibit drug resistance.

Apart from miRNA, long non-coding RNA (lncRNA) also plays a crucial role in suppressing drug tolerance by reducing the effect of RBPs. In a clinical study involving non-small cell lung carcinoma (NSCLC) patient samples, FENDRR expression was downregulated. Overexpression of FENDRR hampered multidrug resistance 1 (MDR1) gene expression through competitive interaction with the 3′UTR of MDR1, thereby hindering HuR expression. This in turn attenuated cancer stemness (Gong et al., 2019). LncRNA-mediated disruption of the HuR/MDR1 axis may significantly contribute to the reduction of chemoresistance failures. On the other hand, clinical research by the Deng group exhibited that the interplay between lncRNA CCAL and cytoplasmic HuR stabilized the CTNNB1 gene, which promoted oxaliplatin resistance in colorectal cancer (Deng et al., 2020). The enzyme cyclooxygenase-2 (COX-2) suppresses the cleavage of both HuR and the pro-apoptotic protein caspase-3, collectively promoting resistance to paclitaxel in oral cancer cells. Moreover, in oral squamous cell carcinoma, the levels of cleaved HuR were lower compared to the full-length HuR, which enhanced cancer proliferation by extending the half-life of COX-2 mRNA transcripts (Janakiraman et al., 2017). In another study, silencing of HuR combined with epirubicin treatment on a colon cancer cell line resulted in the suppression of drug resistance genes like MRP1 and P-gp at both mRNA and protein levels. This study also showed decreased expression of the anti-apoptotic gene BCL2 and increased levels of the pro-apoptotic genes BAX and caspase. The Lin group’s findings demonstrated that HuR suppression enhanced epirubicin sensitivity in colon cancer cell lines *in vitro*, suggesting its potential as a combinatorial treatment to reverse chemotherapeutic drug resistance. In addition, HuR downregulation impedes β-catenin signaling, which is involved in cancer stem cell self-renewal and migration (Lin et al., 2017). Notably, when HuR was knocked down, the expression of the snail protein also decreased, resulting in enhanced tumor response to cisplatin in lung carcinoma cells (Zhou et al., 2016). Importantly, HuR silencing improved the sensitivity of chemotherapeutic drugs such as etoposide, topotecan, and cisplatin in the U251 glioma cell line (Filippova et al., 2011). While several pathways contribute to drug resistance in various cancer types, HuR overexpression is a constant. Figure 3 gives a summary of all the HuR-mediated pathways that contribute to chemoresistance.

#### NONO

**FIGURE 4:**
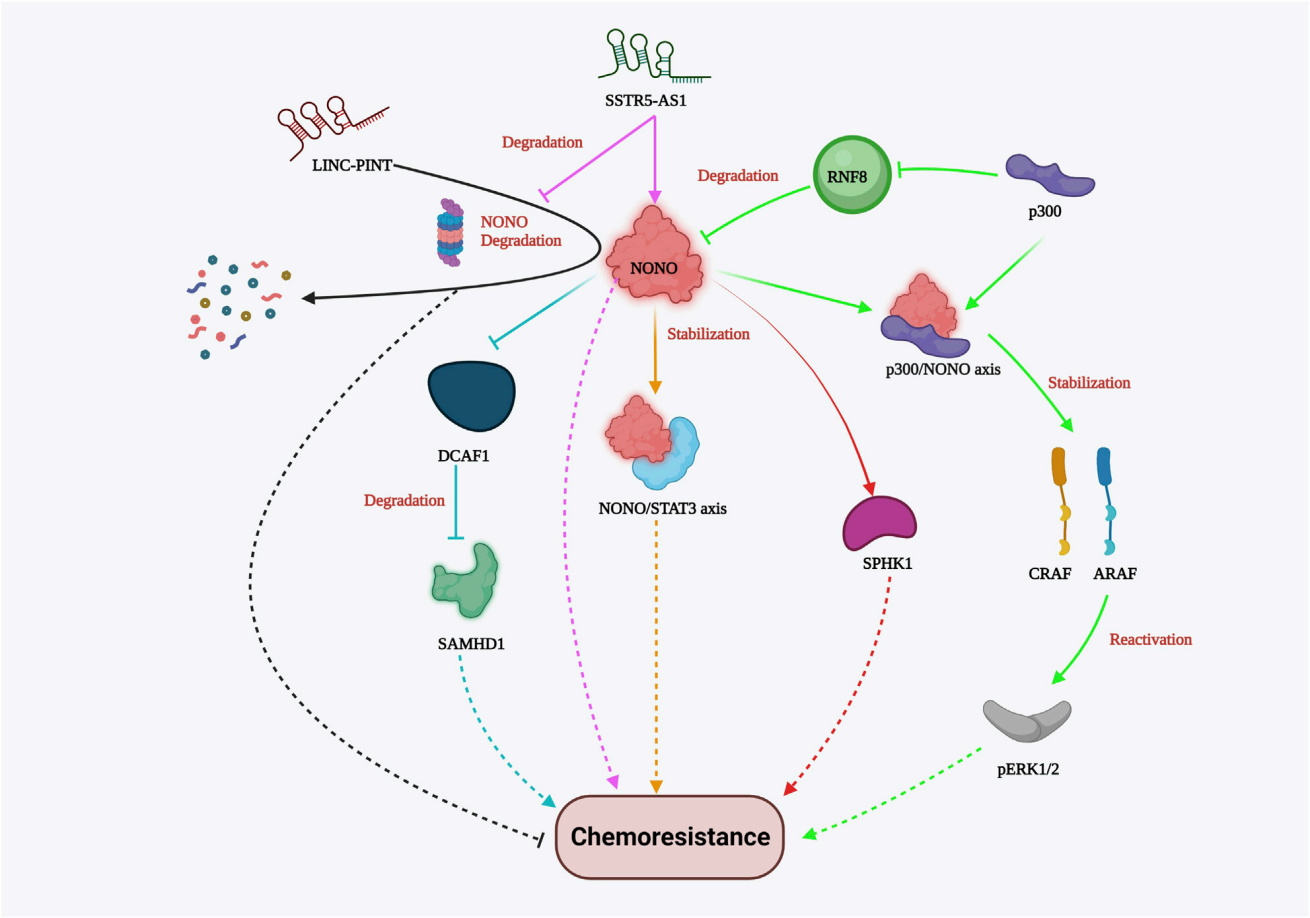
NONO-mediated drug resistance pathway. NONO mediated drug resistance includes stabilization of NONO/STAT3 axis, p300/NONO axis, SPHK1, CRAF, ARAF, SAMHD1; reactivation of Perk1/2; DCAF1 inhibition; lncRNA SSTR5-AS1 stabilizes NONO whereas LINC-PINT and RNF8 promote NONO degradation resulting in chemosensitivity.

NONO is an octamer-binding protein with a non-POU domain that plays various molecular roles, including DNA damage repair, apoptosis, and proliferation, in multiple cancers. It is typically localized in the mammalian nucleus and dispersed throughout the sub-nuclear region. In triple-negative breast cancer (TNBC), NONO is overexpressed compared to normal breast cancer cells. It promotes cancer progression and growth by stabilizing the transcription factor signal transducer and activator of transcription 3 (STAT3). NONO not only binds with STAT3 mRNA but also co-immunoprecipitates with the STAT3 protein, thereby enhancing protein stability and activity. Drug resistance to doxorubicin was greatly influenced by NONO and STAT3 gene expression, which was evident by the CCK8 cell viability assay and clonogenic assay (Kim et al., 2020). In addition, the NONO/STAT3 axis also regulates SPHK1-mediated cisplatin resistance in bladder cancer cells (Qin et al., 2021). NONO also modulates the expression of SAMHD1 (an adenosine triphosphate hydrolase) directly by binding to its RNA recognition motif (RRM). Studies have shown that NONO prevents DCAF1-mediated SAMHD1 degradation, thereby increasing SAMHD1 stability. Silencing NONO was found to increase the sensitivity of acute myelogenous leukemia (AML) cells to cytarabine (Ara-C), as demonstrated by cell proliferation and Annexin V apoptosis assays (Zhang et al., 2022a). In colon cancer, Y-box binding protein 1 (YB-1) contributes to oxaliplatin resistance, and this resistance depends on NONO. In colorectal tumors overexpressing the YB-1 protein, inhibiting NONO or RALY substantially reduced oxaliplatin resistance (Tsofack et al., 2011). Notably, p300 acetyltransferase hinders RNF8-induced NONO degradation in drug-resistant melanoma cells. The NONO/p300 axis contributes to CRAF and ARAF stability, thereby reactivating pERK1/2 (kinase) signaling and ultimately reducing the effectiveness of BRAF inhibitors such as AZ628 and Vemurafenib (RAF kinase inhibitor) (Zhang et al., 2021a). These studies strongly suggest that NONO plays an important role in chemoresistance by providing stability to other proteins that favor cancer cell viability and proliferation. Furthermore, lncRNAs play a role in modulating NONO-mediated drug resistance both favorably and adversely. For instance, the importance of lncRNA, a long intergenic non-protein coding RNA, p53 induced transcript (LINC-PINT), has been highlighted by Chen’s group demonstrating its antagonistic effect against NONO in Paclitaxel resistant TNBC cells. LINC-PINT interacts with NONO, enhancing its proteasome-mediated destruction (Chen et al., 2020). On the other hand, in gallbladder carcinoma (GBC), lncRNA SSTR5-AS1 resists proteosome-mediated NONO degradation and amplifies gemcitabine resistance by stabilizing NONO (Xue et al., 2020). Although the link between lncRNAs and NONO varies between cancer types, lncRNAs hold significant potential for cancer specific NONO targeted therapy for reducing chemoresistance. Overall, these investigations elucidated the significance of the NONO protein in the emergence of chemoresistance (Figure 4). NONO’s multifaceted molecular roles and its interactions with various cellular components underscore its importance as a potential therapeutic target to overcome drug resistance in cancer.

#### IGF2BP2

**FIGURE 5:**
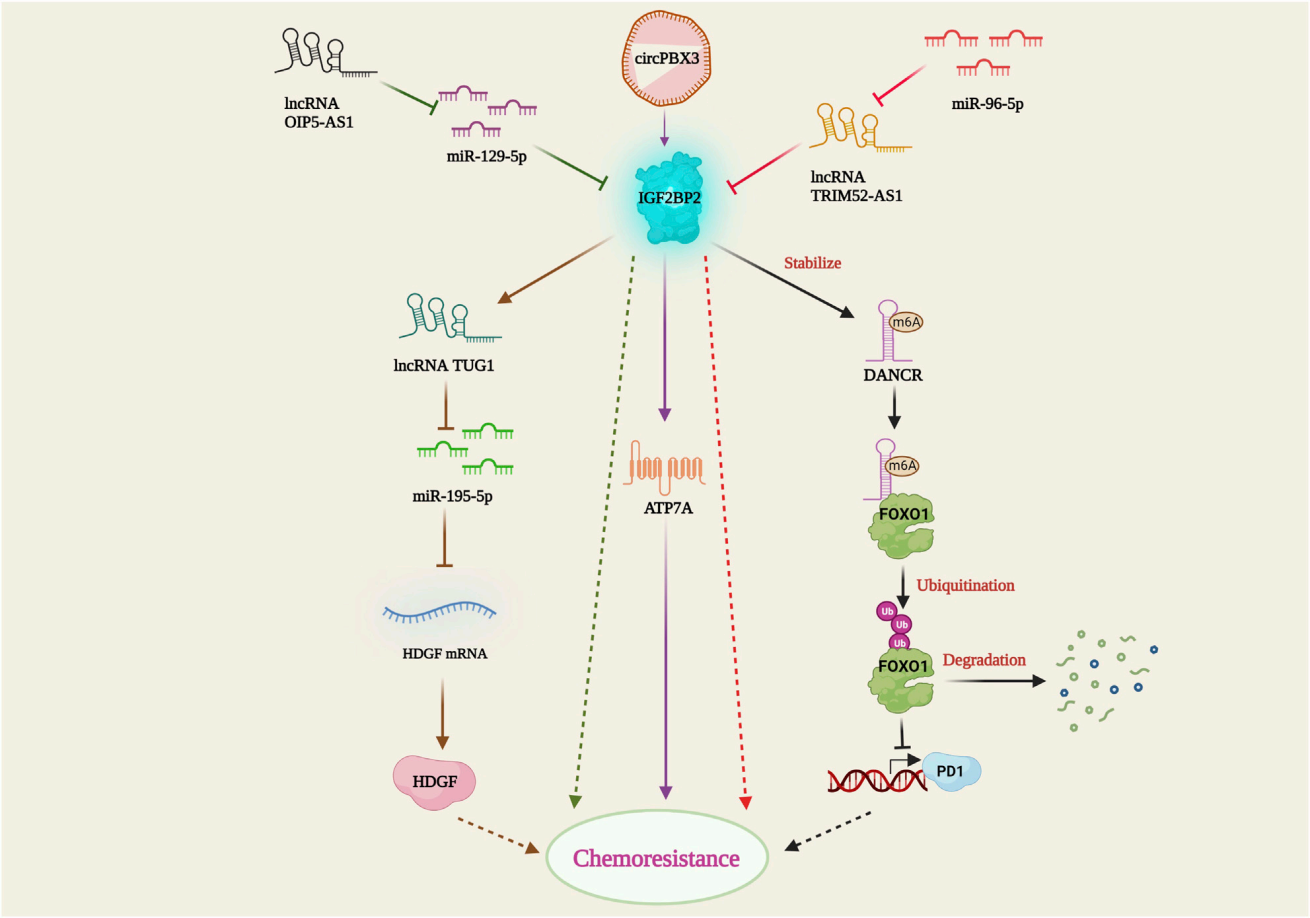
IGF2BP2-mediated drug resistance pathway. IGF2BP2 bring about resistance by stabilizing lncRNA DANCR; inhibition of FOXO1 mediated PD1 expression; lncRNA TUG1 activation; TUG1 mediated miR-195-5p suppression; HDGF activation; ATP7A stabilization through circPBX3/IGF2BP2 axis. lncRNA OIP5-AS1 and miR-96-5P hamper miR-129-5p and lncRNA TRIM52-AS1 facilitated IGF2BP2 suppression.

Insulin-like growth factor 2 mRNA binding protein 2 (IGF2BP2) is an RNA-binding protein that plays several important biological roles in disease metabolism and cancer. It predominantly recognizes and binds to the N6-methyladenosine (m6A) post-transcriptionally modified mRNA, thereby regulating its stability and gene expression. IGF2BP2 is highly expressed in certain cancer types and promotes cancer progression (Wang et al., 2021). Silencing of IGF2BP2 has been shown to enhance apoptosis and downregulate mesenchymal markers such as vimentin, N-cadherin, and snail, leading to the suspension of epithelial-mesenchymal transition (EMT) and ultimately resulting in increased sensitivity of non-small cell lung carcinoma cells to pemetrexed (PME) (Han et al., 2021). In leukemia, IGF2BP1 increases the stemness of leukemia cells by positively regulating the hematopoietic stem cell regulators HOXB4 and MYB, as well as the stem cell marker ALDH1A1. Restricting IGF2BP1 expression resulted in increased sensitivity of leukemia cells to doxorubicin, cytarabine, and cyclophosphamide (Elcheva et al., 2020), showing promising potential for chemoresistance therapy. Furthermore, the interaction between IGF2BP2 and several lncRNAs and miRNAs in cancer cells has been studied by multiple research groups. IGF2BP2 has been shown to interact with and stabilize the methylated DANCR lncRNA, thereby inhibiting Forkhead box protein O1 (FOXO1)-mediated phosphotyrosine interaction domain-containing 1 (PID1) expression. This IGF2BP2/DANCR-mediated suppression of FOXO1 led to etoposide resistance in glioblastoma cells (Han et al., 2022). In colon cancer cells, mir-195-5p binds to the hepatoma-derived growth factor (HDGF) and suppresses its expression, promoting cisplatin-induced apoptosis. However, IGF2BP2-strengthens the lncRNA Taurine upregulated gene 1 (TUG1), which further binds and represses mir-195-5p, thereby facilitating HDGF-mediated cisplatin resistance (Xia et al., 2022). Moreover, miR-129-5p downregulates IGF2BP2 expression, inhibiting cell proliferation and enhancing apoptosis. On the contrary, lncRNA OIP5 antisense RNA 1 (OIP5-AS1) interferes with miR129-5p mediated IGF2BP2 suppression, resulting in increased IGF2BP2 expression and temozolomide (TMZ) resistance in glioblastoma cells (Wang et al., 2022). Surprisingly, miR-96-5p follows a unique chemoresistance pathway, where it impedes lncRNA TRIM52-AS1 and induces IGF2BP2-mediated CDDP resistance in cervical cancer cell lines (Wu et al., 2022). In ovarian cancer, the circular RNA circPBX3 directly attaches to IGF2BP2 and stabilizes the copper transporter gene ATP7A, thereby conferring cisplatin tolerance (Fu et al., 2022). The above-mentioned studies emphasized the critical relationship between IGF2BP2 and ncRNAs in establishing drug resistance (Figure 5). Understanding these interactions may offer potential therapeutic targets to overcome drug resistance in cancer and improve treatment outcomes.

#### IGF2BP3

IGF2BP3, an oncofetal RNA binding protein, contributes to tumor progression, growth, and chemoresistance (Lederer et al., 2014). The Xu study group found that among 151 colon cancer patients who participated in clinical trials, IGF2BP2 expression was high in 57% of the cases (Xu et al., 2019). This is corroborated by the fact that enhanced IMP3 expression was found in over 58% of end-stage renal cell carcinoma tumors (stages III and IV), and more than 40% in hepatocellular carcinoma (HCC) (Li et al., 2015; Pei et al., 2015). Furthermore, in HCT116 and LoVo colorectal cancer cells, silencing IGF2BP2 led to decreased cell proliferation, colony formation, migration, and a greater proportion of cells in the G0/G1 phase of the cell cycle. In animal models, IGF2BP2 knockdown also decreased the tumor burden (Xu et al., 2019). This study highlighted the vibrant role of IGF2BP3 in cancer progression. An *Invitro* investigation revealed that IMP3 positively stabilized the expression pattern of ABCG2, a key drug efflux protein, promoting doxorubicin and mitoxantrone resistance in triple-negative breast cancer (Samanta et al., 2013). Furthermore, via stabilizing COX6B2 and boosting oxidative phosphorylation, IGF2BP3 confers resistance to tyrosine kinase inhibitors in NSCLC (Lin et al., 2023). Similarly, IGF2BP3 overexpression de-sensitized sorafenib and doxorubicin by enhancing cancer stem cell marker CD133 and drug efflux proteins such as ABCB1 and ABCG2 in human hepatocellular carcinoma. On the brighter side, drug resistance in HCC was suppressed by isocorydine derivative (d-ICD) as it downregulated drug efflux protein and IGF2BP3 (Li et al., 2015; Mancarella and Scotlandi, 2020). These studies firmly imply that downregulation of IGF2BP3 could be a promising approach to overcome drug resistance in colon cancer and hepatocellular carcinoma.

#### Musashi-1

**FIGURE 6:**
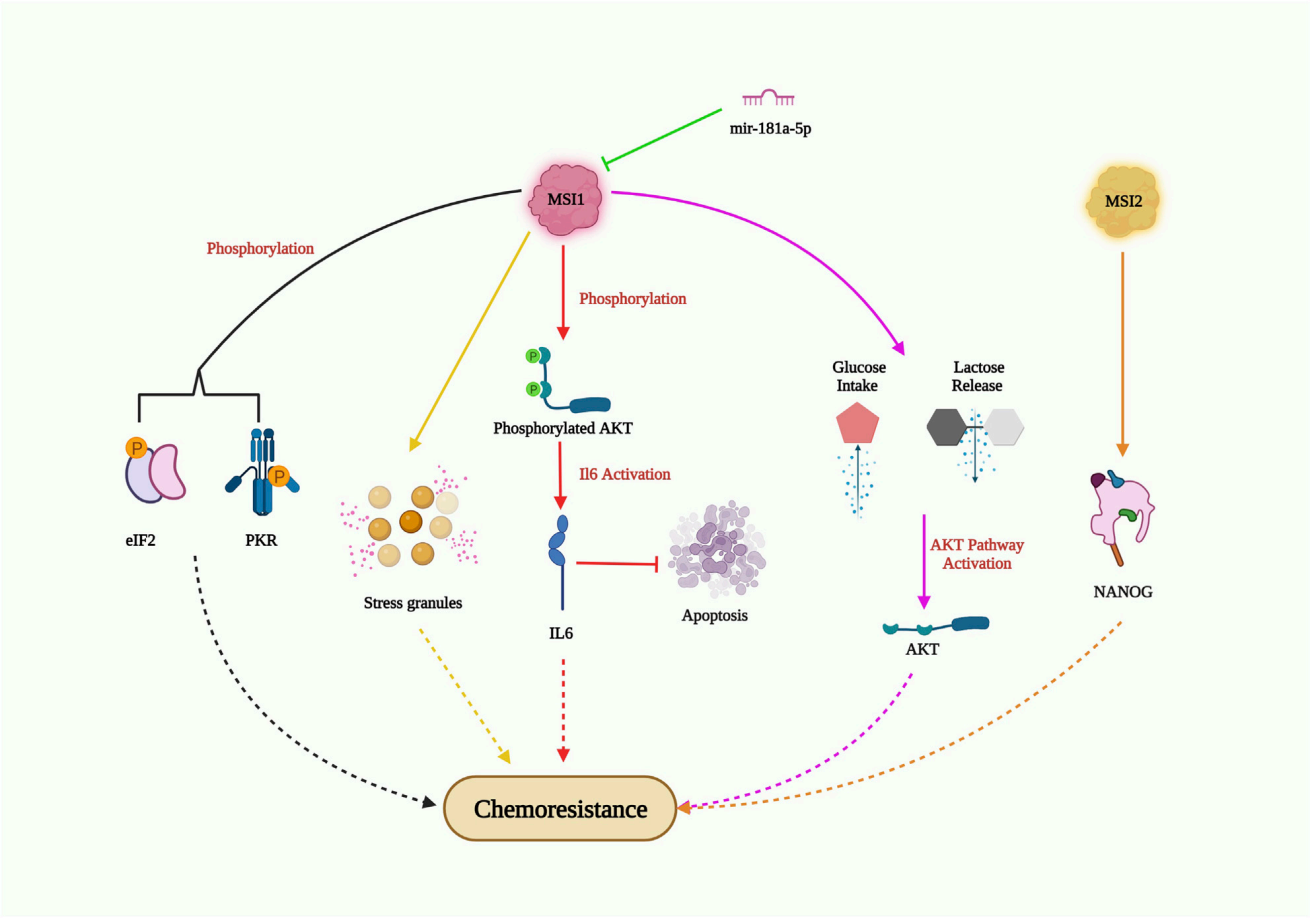
MSI-mediated drug resistance pathway. MSI1 enters chemoresistance by AKT phosphorylation; IL6 activation and suppression of apoptosis; AKT pathway activation through high glucose intake and lactose release; generation of stress granules; phosphorylation of EIF2 and PKR; MS12 also induce chemoresistance by elevating NANOG expression in CSC; however, miR-181a-5p obstructs MSI1 expression and could inhibit drug resistance.

Musashi (MSI), a neuronal RNA-binding protein, was discovered to play a significant part in asymmetric division and extrasensory organ development in adult *Drosophila* (Nakamura et al., 1994). MSI1 and MSI2 are two distinctive homologs of Musashi (Sakakibara et al., 2001). Being a crucial stem cell molecule, MSI recognizes (G/A) Un (AGU) sequence motifs (*n* = 1–3) at the 3′UTR of its target mRNA and blocks its translation (Okabe et al., 2001). The overexpression of MSI1 has been observed in multiple malignancies, including glioblastoma, lung, breast, and colon cancer, and has been reported to promote tumor growth and recurrence (Forouzanfar et al., 2020). Several elaborate studies on the relationship between MSI1 and drug resistance (Figure 6) have been performed on glioblastoma (GBM) cells. In GMB cells, MSI1 overexpression led to improved cell survival and maintenance of tumorigenesis when exposed to the chemotherapeutic agent cisplatin. MSI1 promoted AKT phosphorylation, which positively regulated IL-6 secretion, thereby attenuating the chemotherapeutic-induced apoptotic pathway (Chen et al., 2016). In pediatric GBM cells with elevated MSI1 expression, greater resistance to the combined drug treatment of temozolomide and valproic acid was observed, suggesting the potential to increase drug sensitivity by impairing MSI1 along with combinatorial drug treatment (Pötschke et al., 2020). In colorectal cancer, MSI1 overexpression resulted in the formation of stress granules (SGs), which progressively increased resistance to 5-FU treatment. The C-terminal region of MSI1 contributed significantly toward synthesizing 5-FU-activated SGs, contributing to the prevention of 5-FU-mediated apoptosis (Chiou et al., 2017). However, Chen et al. revealed that both the C and N-terminus of MSI1 were critical for SG synthesis. In glioblastoma cells with MSI1 overexpression treated with arsenic trioxide (ATO), MSI1 harbored and phosphorylated protein kinase R (PKR) and eukaryotic initiation factor 2 (eIF2), resulting in activation of the PRK/eLF2 signaling cascade, increased cancer stem cell signature expression, and drug resistance (Chen et al., 2018). Interestingly, enhanced MSI1 expression increased glucose uptake and lactate release in cisplatin (CDDP)-resistant NSCLC cell lines A549 and H522. It also decreased CDDP sensitivity to NSCLC through the AKT-mediated pathway. As for the relationship between MSI1 and miRNAs, a study showed that miR-181a-5p targets MSI1 and negatively correlates with MSI1 (Lang et al., 2017). These studies highlight the critical role of MSI1 overexpression in drug resistance and its potential as a therapeutic target to overcome drug resistance in various cancers. Understanding the mechanisms of MSI1-mediated drug resistance could provide valuable insights for the development of more effective treatment strategies.

#### Musashi-2

Musashi-2 (MSI2), an RNA-binding protein, is known to be overexpressed and plays a critical role in enhancing cancer stemness and drug resistance in NSCLCs (Figure 6). In PC9 cells (EGFR tyrosine kinase sensitive cells), MSI2 overexpression decreased the sensitivity to kinase inhibitors such as gefitinib or osimertinib. Additionally, MSI2 upregulation positively regulated NANOG expression, thereby enhancing cancer stem cell characteristics (Yiming et al., 2021). In acute myeloid lymphoma cells, MSI2 knockdown increased the sensitivity to daunorubicin and triggered apoptosis, suggesting its role in promoting drug resistance in this context (Han et al., 2015). Fang’s group further demonstrated the importance of MSI2 in cancer stem cell maintenance and chemoresistance. They found that increased expression of MSI2 was observed in CD133^+^ or OV6^+^ hepatocellular carcinoma stem cell populations, along with elevated expression of pluripotency factors. MSI2 silencing led to reduced cell survival, colony numbers, and increased apoptosis rates, resulting in improved sorafenib sensitivity (Fang et al., 2017). Overall, MSI2 overexpression increases cancer cell stemness and viability, highlighting its importance as a potential target for overcoming drug resistance and reducing cancer cell stemness in NSCLCs and other cancer types.

#### LIN28

**FIGURE 7:**
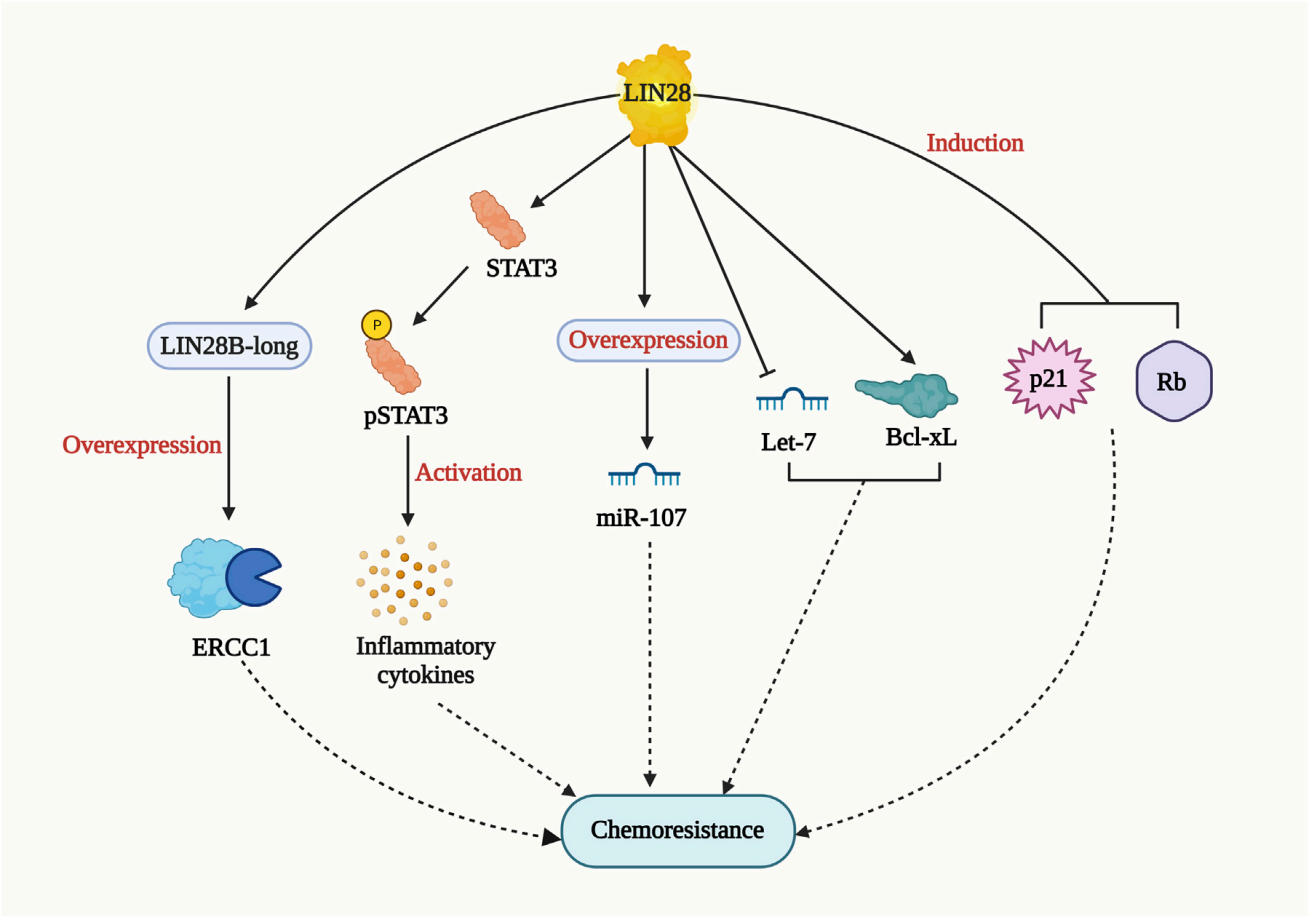
LIN28-mediated drug resistance pathway. LIN28 induce drug resistance by multiple mechanisms including Lin28/let-7/Bcl-xl; Lin28/STAT3 signaling pathway; p21 and RB induction; LIN28B-long/ERCC1/let-7 pathway.

Lin28 is an RNA-binding protein identified in *Caenorhabditis elegans* as a regulator of developmental control. Subsequently, it has been found to play essential roles in stem cell differentiation, glucose metabolism, normal development, and cancer (Thornton and Gregory, 2012). In gastric cancer cell lines, Lin28 overexpression was shown to increase chemoresistance to oxaliplatin, doxorubicin, paclitaxel, and 5-fluorouracil when compared with control cells. On the other hand, the knockdown of Lin28 led to a reduction in the drug-resistance ability. The study revealed that Lin28 targets miR-107, which is involved in the chemo-resistance mechanism (Teng et al., 2015). In breast cancer cell lines, higher Lin28 expression was associated with resistance to paclitaxel. Knockdown of Lin28 in the T47D cell line restored paclitaxel sensitivity. The study also suggested that induction of p21 and Rb expression and inhibition of Let-7 miRNA levels could be possible mechanisms of chemoresistance (Lv et al., 2012). In hepatocellular carcinoma (HCC), Lin28 has been linked to tumor relapse after chemotherapy. A drug resistant Hep3B cell line was found to express more Lin28 and exhibit greater resistance to paclitaxel and other anti-cancer drugs compared to the parental Hep3B cell line. The Lin28/let-7/Bcl-xl pathway was proposed as a drug resistance mechanism in the Hep3B cell line (Tian et al., 2014). Additionally, Lin28 promotes cisplatin and radio resistance in NSCLC via adversely regulating let7 miRNA (Yin et al., 2017). These findings strongly imply that addressing the Lin28/Let7 axis may help prevent tumor progression and restore drug sensitivity in cancer cells. Lin28B, which has two isoforms (Lin28-short and Lin28-long), is involved in multiple drug resistance mechanisms (Figure 7). In colon cancer, the Lin28 null cell line and Lin28B-long isoform-expressing cells demonstrated a significant increase in drug resistance compared to the Lin28 null cell line. The overexpression of excision repair cross-complementing group 1 (ERCC1) in the Lin28B-long isoform expressing cell line contributed to chemo-resistance through a let-7 dependent mechanism (Mizuno et al., 2018). Moreover, in a cholangiocarcinoma (CAA) cell line called MMNK-1 cells, Lin28B-overexpression led to greater chemotherapeutic resistance compared to the control cells. Modulation of the Lin28/STAT3 signaling pathway was implicated in inducing cholangiocytes, contributing to CAA initiation (Puthdee et al., 2021). These studies collectively demonstrate the significant role of Lin28 in promoting drug resistance in various cancer types. As mentioned earlier, STAT3 has also been connected to NONO and has been shown to induce cancer cell chemoresistance. The relationship between Lin28/NONO/STAT3 could provide promising insights into cancer cell chemoresistance mechanisms.

### RNA binding motif (RBM) proteins

RBPs with distinct domains, including ribonucleoproteins (RNPs), cold-shock domains (CSDs), and zinc fingers (ZnFs), among others, are collectively referred to as RBM proteins. RBM proteins often contain one or more RNA recognition motifs (RRMs); for example, RBM3 possesses one RRM, and RBM19 contains six RRMs (Li et al., 2021). The RBM protein family plays critical roles in various biological activities such as RNA stability, mRNA translation, metabolism, and pre-mRNA splicing. (Lee et al., 1996; Dreyfuss et al., 2002; Maris et al., 2005; Jonas et al., 2020; Van Nostrand et al., 2020). In the context of cancer, RBM have been shown to impact chemotherapeutic drug sensitivity and drug resistance. For example, silencing RBM3 in bladder cancer led to reduced sensitivity to cisplatin and gemcitabine (Wahlin et al., 2022). In breast cancer cells, RBM6 deficiency increased sensitivity to ionizing radiation (IR), PARP inhibitors (PARPi), ATM inhibitors (ATMi), and cisplatin. RBM6 expression promoted homologous recombination repair in double-strand breaks, contributing to altered chemotherapeutic drug sensitivity (Machour et al., 2021). Increased expression of RBMx was observed in hepatocellular carcinoma (HCC) patient tissue and HCC cell lines. RBMx overexpression was associated with enhanced cell viability, proliferation, and resistance to sorafenib. The long non-coding RNA bladder cancer-associated transcript 1 (LncBLACAT1) was identified as a stabilizer of RBMx and was found to promote HCC development and drug resistance (Song et al., 2020). In small-cell lung cancer cell lines, RBM5 expression was shown to slow cell growth and increased sensitivity to cisplatin (Loiselle et al., 2016). In ADM-resistant breast cancer tissues and the ADM-resistant MCF-7/A cell line, RBM38 expression was significantly higher. The interaction between RBM and the miRNA miR-320b in cancer cell chemoresistance has also been highlighted. In a study, miR-320b was found to reduce ADM resistance and increase sensitivity in MCF7/A cell lines by suppressing RBM38 expression. RBM38 protected against miR-320b-induced chemosensitivity by regulating proteins involved in apoptosis, drug resistance, and cell cycle progression (Ke et al., 2022). The miRNA miR-320b also holds promise as a potential target to regulate RBM and increase chemosensitivity in cancer cells. These findings highlight the crucial roles of RBM in cancer drug resistance and sensitivity, which may facilitate overcoming chemoresistance and improve treatment outcomes in various types of cancer.

#### Pumilio

RNA-binding protein Pumilio1 (PUM1) belongs to the highly conserved PUF (Pumilio-Fem3-binding Factor) family. The human PUM1 gene is 5,385 bp long, consisting of 22 exons, and codes for 1,188 residues (127 kDa). PUM1 plays a crucial role in the post-transcriptional regulation of mRNA targets by binding to the 3′UTR consensus sequence 5′-UGUANAUA-3′ and is mainly located in the cytoplasm (Gor et al., 2021). In colorectal cancer, *in silico* analysis using the Human Protein Atlas and Cancer Genome Atlas revealed elevated levels of both PUM1 protein and mRNA transcripts. PUM proteins have contradictory roles; in some cases, they support cancer cell growth and proliferation, while in others, they are reported to repress oncogenes in various cancers (Gong et al., 2022). A recent study by Gor et al. reported that PUM1 overexpression promoted tumor cell proliferation and migration in colon cancer cell lines, and elevated levels of PUM1 were observed in primary and metastatic colon cancer cell lines (Gor et al., 2021). PUM1 is a key regulator for the initiation and progression of colorectal cancer. P21, a cell cycle-associated gene and a negative regulator of the cell cycle, is a direct mRNA target of PUM1. Knockout or knockdown of PUM1 led to increased p21 expression at both mRNA and protein levels, and it blocked the G1/S phase transition of cells. PUM1 also binds and phosphorylates p27 (tumor suppressor gene) mRNA at the 3′-UTR, promoting a conformational change that exposes the p27 transcript for miRNA-221 and miRNA-222 mediated suppression (Gong et al., 2022). Upregulation of PUM1 in prostate cancer resulted in translational repression of cyclin-dependent kinase inhibitor 1B (CDKNBI), contributing to neoplastic pathogenesis (Gor et al., 2021). Recently, research showed that the flavonoid morin has an affinity for PUM1 and substantially suppresses its expression, reducing the number of cancer stem cells (Gor et al., 2022). On the other hand, the Pumilio homolog, PUM2, has been shown to confer chemotherapeutic drug resistance. Exosome-derived miRNA-130a from cancer-associated fibroblasts (CAFs) can navigate into NSCLCs and induce cisplatin resistance. PUM2 is responsible for packaging this miRNA into the exosomes of CAFs (Zhang et al., 2021b). These findings underscore the intricate roles of PUM1 and PUM2 in cancer development and chemotherapeutic drug resistance and highlight the role of cell-to-cell communication in promoting cancer chemoresistance through exosome trafficking.

#### YTHDF1

YTH N6-methyladenosine RNA binding protein 1 (YTHDF1) is an RNA binding protein that regulates post-transcriptional modification and translation by binding to N6-methylated adenosine. Elevated expression levels of YTHDF1 have been reported in gastric, colon, and breast cancer (Chen et al., 2021a; Chen et al., 2021b; Sun et al., 2022). In breast cancer cell lines (MCF7 and MDA-MB-231), silencing of YTHDF1 increased sensitivity to Adriamycin, cisplatin, and olaparib, highlighting the significance of YTHDF1 in drug resistance and DNA damage repair. YTHDF1, along with METTL14, contributes to the increased stability of E2F8 mRNA, thereby participating in YTHDF1-mediated chemoresistance (Sun et al., 2022). Additionally, YTHDF1 directly targets GLS1, and its activation enhances glutaminase-1 metabolism, promoting cisplatin resistance (Chen et al., 2021a). These findings suggest that YTHDF1 plays a critical role in regulating drug resistance in various cancer types, and miRNA-136-5p could be a promising target to regulate YTHDF1.

#### AUF1

The AU-rich binding factor 1 is a well-known adenylate-uridylate-rich element (ARE)-specific RNA binding protein (ARE-BP) that has been implicated in the dysregulation of various human malignancies. In HCC, AUF1 was found to be highly expressed in HCC tissues, and its overexpression was facilitated by the transcription factor E2F1. AUF1 binds to the 3′UTR of aldo-keto reductase family 1 member B10 (AKR1B10) and enhances its mRNA stability and gene expression, acting as a catalyst for AUF1. In hepatoma cells, overexpression of both AUF1 and AKR1B10 led to increased resistance to doxorubicin (Zhang et al., 2022b). Additionally, AUF1 has been reported to induce resistance to cisplatin and docetaxel in breast stromal fibroblasts (Al-Tweigeri et al., 2022).

These findings highlight the role of AUF1 in promoting drug resistance in cancer cells. Knockdown of AUF1 could be a potential treatment approach to increase drug sensitivity in cancer cells.

### Future perspective

Numerous researchers worldwide have elucidated the pivotal role of RNA binding proteins (RBPs) in the emergence of chemoresistance to current chemotherapeutic agents. Investigations reveal that the intricate interplay between non-coding RNA (ncRNA) and RBPs significantly contributes to chemotherapeutic tolerance. Several studies indicate that the silencing of RBPs promptly hampers the fundamental hallmarks of cancer progression, including cell migration, proliferation, cancer stemness, and induce apoptosis. On a positive note, deciphering the targets associated with RBP-mediated chemoresistance across various malignancies could pave the way for the development of tumor-specific drug combinations. RBPs are crucial in maintaining CSC characteristics, which are key to cancer progression, relapse, and chemoresistance (Phi et al., 2018; Zhou et al., 2021). An inhibitor for Lin28 has been identified and has shown effectiveness in reducing tumor-sphere formation and inducing differentiation (Roos et al., 2016). Additionally, several other reports have highlighted inhibitors for MS1, PUM1, and HuR, among others, resulting in decreased RNA-binding activity. This reduction has been associated with a significant decrease in cancer progression and the maintenance of cancer stem cells (Meisner et al., 2007; Minuesa et al., 2019; Gor et al., 2022; Gor et al., 2023). Small interfering RNA (siRNA) and small molecule inhibitors exhibited promising effects in impeding the interaction between RBPs and their targets. For instance, the small molecule inhibitor AZA-9 demonstrated notable binding to the RNA recognition motif, effectively disrupting the interconnection between HUR and target RNAs (Kaur et al., 2017). Similar observations were made in LIN28, where the small molecule inhibitor LI71 hindered the LIN28/let-7 interaction by binding to its cold shock domain (Wang et al., 2018). In essence, understanding the intricate mechanisms of drug resistance associated with RBPs holds the potential to pave the way for innovative and targeted therapies in cancer treatment. The recent therapeutic applications of targeting RBPs in cancer, such as RNA vaccines for tumor-specific antigens, antisense oligonucleotides (ASOs) (Xiong et al., 2021), to alter the gene expression of cancer related genes, and aptamers (Kim et al., 2021b), to disrupt cancer pathways, have been explored. These studies are critical for assessing the therapeutic potential and safety of these novel treatments. The dysregulation of RBPs is intricately linked to cancer chemoresistance. Understanding these molecular mechanisms opens new possibilities for targeted cancer therapies. A personalized approach, considering specific cancer types and individual patient profiles, could lead to more effective treatments and reduced chemoresistance. The future of cancer therapy may well hinge on the strategic targeting of RBPs and their associated pathways.

## Conclusion

RBPs play a critical role as evolutionarily conserved master regulators of post-transcriptional gene modification, influencing RNA stability, metabolism, pre-mRNA splicing, and mRNA translocation. Dysregulation of RBPs has been shown to play an important role in drug resistance and has been extensively studied in various cancer types, including breast, colon, gastric, and HCC. This comprehensive review sheds light on the potential mechanisms of cancer chemoresistance in which RBPs participate, their interaction with proteins that promote cancer cell stemness and chemoresistance, and their link to ncRNAs, providing valuable insights for researchers and aiding in the selection of targets as well as the development of better diagnostic and treatment strategies for cancer patients.

## Figures and Tables

**Table:** 

**5-FU**	5-fluorouracil
**ABCG2**	ATP Binding Cassette Subfamily G Member 2
**ADM**	Adriamycin
**AKR1B10**	Aldo-keto reductase family 1 member B10
**AML**	Acute myelogenous leukemia
**Ara-C**	Cytarabine
**ARE**	Adenine and uracil-rich elements
**ATMi**	ATM inhibitor
**ATO**	Arsenic trioxide
**ATP7A**	ATPase copper transporting alpha 7 A
**AUF1**	AU-rich binding factor 1
**BAX**	Bcl-2-associated X protein
**Bcl-xL**	B-cell lymphoma-extra large
**CAA**	Cholangiocarcinoma
**CAF**	Cancer-associated fibroblasts
**CDC6**	Cell division cycle 6
**CDDP**	Cisplatin
**CDKNBI**	Cyclin-dependent kinase inhibitor 1B
**COX-2**	Cyclooxygenase-2
**CSC**	Cancer stem cell
**CSD**	Cold-shock domain
**CSTF2**	Cleavage stimulation factor subunit 2
**DANCR**	Differentiation antagonizing non-protein coding RNA
**DTX**	Docetaxel
**EGFR**	Epidermal growth factor receptor
**eIF2**	Eukaryotic translation initiation factor 2A
**ELAV**	Embryonic lethal abnormal vision
**EMT**	Epithelial to mesenchymal transition
**ERCC1**	Excision repair cross-complementing group 1
**FOXO1**	Forkhead box protein O1
**GBC**	Gallbladder carcinoma
**GBM**	Glioblastoma
**GLS1**	Glutaminase-1
**HCC**	Hepatocellular carcinoma
**HDGF**	Hepatoma-derived growth factor
**HNS**	HuR nucleocytoplasmic shuttling sequence
**HOXB4**	Homeobox B4
**HUR**	Human antigen R
**IGF2BP2**	Insulin-like growth factor 2 mRNA binding protein 2
**IL-6**	Interleukin 6
**IR**	Ionizing radiation
**LncBLACAT1**	Long non-coding RNA bladder cancer-associated transcript 1
**L-OHP**	Oxaliplatin
**m6A**	N6-methyladenosine
**METTL14**	Methyltransferase-like 14
**miRNA**	MicroRNA
**MRP1**	Multidrug resistance-associated protein 1
**MSI**	Musashi
**ncRNA**	Non-coding RNA
**NES**	Nuclear export signal
**NLS**	Nuclear localization signals
**NONO**	Non-POU Domain Containing Octamer Binding
**NSCLC**	Non-Small Cell Lung Carcinoma
**PARPi**	PARP inhibitor
**P-gp**	P-glycoprotein
**PID1**	Phosphotyrosine interaction domain containing 1
**PKR**	Protein kinase R
**PUF**	Pumilio-Fem3-binding Factor
**PUM**	Pumilio
**RBM**	RNA binding motif
**RBP**	RNA binding protein
**RNPs**	Ribonucleoproteins
**RRM**	RNA recognition motifs
**SG**	Stress granules
**STAT3**	Signal transducer and activator of transcription 3
**TAMR**	Tamoxifen-resistant
**TMZ**	Temozolomide
**TNBC**	Triple-negative breast cancer
**TUG1**	Taurine upregulated gene 1
**UTR**	Untranslated regions
**YB-1**	Y-box binding protein 1
**YTHDF1**	YTH N6-methyladenosine RNA binding protein 1
**ZnF**	Zinc finger
